# Combination therapy targeting the elevated interleukin‐6 level reduces invasive migration of BRAF inhibitor‐resistant melanoma cells

**DOI:** 10.1002/1878-0261.12433

**Published:** 2019-01-10

**Authors:** Purusottam Mohapatra, Chandra Prakash Prasad, Tommy Andersson

**Affiliations:** ^1^ Cell and Experimental Pathology Department of Translational Medicine Clinical Research Centre Skåne University Hospital Lund University Malmö Sweden; ^2^ Present address: Department of Medical Oncology (Laboratory) Dr. B.R. Ambedkar IRCH All India Institute of Medical Sciences New Delhi India

**Keywords:** BRAF inhibitor‐resistant, interleukin‐6, invasion, melanoma, migration, WNT5A

## Abstract

The identification of novel antimetastatic therapeutic targets is necessary for improved treatment of patients with acquired BRAF inhibitor‐resistant (BRAFi‐R) melanoma, in whom metastasis is a major concern. Our present study focused on the identification of such targets to explore novel antimetastatic therapeutic options for BRAFi‐R melanoma patients. We confirmed the development of BRAFi resistance in our BRAFi‐treated melanoma cell lines by demonstrating reduced sensitivity to BRAF inhibitors, increased ERK1/2 activity and increased WNT5A expression. Here, we demonstrated for the first time that high secretion of interleukin‐6 (IL‐6) was associated with increased invasive migration of BRAFi‐R melanoma cells. This finding could be readily explained by the increased expression of WNT5A in BRAFi‐R melanoma cells and the presence of an IL‐6/WNT5A positive feedback loop in parental melanoma cells. Surprisingly, however, we found that the IL‐6/WNT5A positive feedback loop present in parental melanoma cells was lost during the development of acquired BRAFi resistance, meaning that IL‐6 and WNT5A signalling were independent events in BRAFi‐R melanoma cells. Despite the absence of an IL‐6/WNT5A loop, we found that both an IL‐6 blocking antibody and the WNT5A antagonist Box5 alone impaired the elevated invasive migration of BRAFi‐R melanoma cells, but combined use of the two was more effective. This impaired invasive migration of BRAFi‐R melanoma cells correlated well with the reduction in Cdc42‐GTPase activity and alterations of the actin cytoskeleton in these cells. In summary, our novel identification of IL‐6 as a key independent promoter of the invasive migration of BRAFi‐R melanoma cells stresses that a combination of a blocking IL‐6 antibody and administration of the WNT5A antagonist Box5 might be an attractive antimetastatic approach for future treatment of BRAFi‐R melanoma patients.

**Abbreviations**
Box5: t‐butyloxycarbonyl‐modified WNT5A‐derived hexapeptide
BRAFi: BRAF inhibitor
BRAFi‐R: BRAF inhibitor‐resistant
IL‐6 Ab: anti‐human interleukin‐6 antibody
IL‐6: Interleukin‐6

## 1 Introduction

The majority of cancers are caused and promoted by either inherited or acquired genetic changes. In melanomas, the ‘V600E’ mutation in the BRAF gene is the most commonly observed mutation. Patients with melanoma bearing such a cancer‐driving mutation can be effectively treated with a BRAF inhibitor (BRAFi), for example, vemurafenib and dabrafenib (Tsai *et al*., 2008). However, resistance to BRAFi frequently develops in BRAF‐treated melanoma patients and is linked to increased dissemination of these tumours (O'Connell *et al*., 2013). The mechanism(s) underlying this increased metastatic behaviour of BRAFi‐resistant (BRAFi‐R) melanomas is not completely understood even though it is an essential target in future effective treatment strategies for these patients.

The WNT family member WNT5A was originally shown to promote melanoma cell migration by Weeraratna and co‐workers (Weeraratna *et al*., 2002). WNT5A was subsequently further investigated for its potential role in the clinical outcome of melanoma, and WNT5A protein expression was shown to predict a shorter recurrence‐free survival in a large cohort of melanoma patients (Da Forno *et al*., 2008). These studies stimulated a search to identify how the WNT5A ligand affects the tumour promoter activities of melanoma cells (Prasad *et al*., 2015). Despite the important role of the WNT5A ligand in melanoma progression, few studies have focused on the mechanism(s) underlying the increased expression of WNT5A in melanoma (Camilli *et al*., 2011; O'Connell *et al*., 2009). However, we identified that at least one such factor, IL‐6, increases melanoma cell expression of WNT5A through a p38‐MAPK signalling pathway (Linnskog *et al*., 2014). This is an interesting observation since IL‐6 has been previously shown to drive melanoma metastasis via its ability to promote melanoma cell invasion (Kushiro *et al*., 2012; Na *et al*., 2013). Furthermore, in a more recent report, we demonstrated that IL‐6 and WNT5A signalling constitute a positive feedback loop that potentiates the migration and invasion of melanoma cells (Linnskog *et al*., 2016).

The WNT5A ligand has also been implicated as a cause of BRAFi‐R in melanoma (Anastas *et al*., 2014; O'Connell *et al*., 2013). In one of these two studies, the authors reported that the increased expression of WNT5A present in BRAFi‐R melanoma induces its effects through the Frizzled‐7 receptor, whereas the authors of a different study performed with a different setup reported that it occurred via the ROR2 receptor (Anastas *et al*., 2014; O'Connell *et al*., 2013). In one of those studies, the authors were also able to restore the sensitivity of BRAFi‐R cells to the BRAF inhibitor PLX4720 by siRNA knockdown of the putative WNT5A receptor ROR2 (O'Connell *et al*., 2013). However, the secretion, signalling and role of IL‐6 have, to the best of our knowledge, not yet been studied in acquired BRAFi‐R melanomas.

In this study, we demonstrate for the first time that the induction of BRAFi‐R in melanoma cells results in a prominent amplification of IL‐6 secretion along with elevated WNT5A expression. Interestingly, the IL‐6/WNT5A positive feedback loop present in parental melanoma cells is lost during the development of BRAFi resistance in melanoma cells, and therefore, IL‐6 and WNT5A signalling cascades in these cells constitute independent regulatory events. The inhibition of either of these independent signalling events impaired the invasive migration of BRAFi‐R melanoma cells, although combined targeting was more effective than targeting each molecule alone to reduce the invasive migration of BRAFi‐R cells, suggesting a possible novel therapeutic approach for the future antimetastatic treatment of BRAFi‐R melanoma patients.

## 2 Materials and methods

### 2.1 Cell culture, reagents and treatments

The *BRAF*‐mutant HTB63 melanoma cell line (Origin: Metastatic site; Common mutations: *BRAF* ^*V600E*^ */NRAS* ^*WT*^ */PTEN* ^*−/−*^ */TP53* ^*WT*^; Cat# ATCC‐HTB‐63), the A375 melanoma cell line (Origin: Metastatic site; Common mutations: *BRAF* ^*V600E*^ */NRAS* ^*WT*^ */PTEN* ^*WT*^ */TP53* ^*WT*^; Cat# ATCC‐CRL‐1619) and the A2058 melanoma cell lines (Origin: Metastatic site; Common mutations: *BRAF* ^*V600E*^ */NRAS* ^*WT*^ */PTEN* ^*−/−*^ */TP53* ^*V274F*^; Cat# ATCC‐CRL‐11147) were all purchased from ATCC and cultured as per the supplier's instruction. HTB63, A375 and A2058 cells were cultured in McCoy's 5A or DMEM supplemented with 10% FBS, 5 U·mL^−1^ penicillin, 0.5 U·mL^−1^ streptomycin and 2 mm glutamine. The genetic authentication of the HTB63, A375 and A2058 cell lines was confirmed by the supplier.

All of the cell lines were routinely serum‐starved in 1% FBS‐supplemented medium for 24 h prior to each experiment. All the cell lines were routinely screened for the absence of mycoplasma. All three BRAFi‐resistant cell lines were properly stored in liquid nitrogen for other experiments. Before and during the experiments, resistance to BRAFi in these cells was assessed using the MTT assay. The cells were maintained in drug‐free medium for a maximum of 10 passages during these experiments.

### 2.2 Establishing PLX4032 (BRAF inhibitor)‐resistant melanoma cell lines

The HTB63, A375 and A2058 melanoma cell lines were treated with 2 μm PLX4032 (Selleckchem, Munich, Germany, Cat# S1267) every third day for 4 weeks. The concentration of PLX4032 was then increased to 5 μm for the next 8 weeks. During these processes, the treatment media were replaced every 72 h with fresh medium containing PLX4032. After 12 weeks, the cell lines were assessed for their resistance to PLX4032 using the MTT cell viability assay. ERK1/2 activity and WNT5A expression levels were also measured. Cell lines exhibiting reduced sensitivity to PLX4032 and increased ERK1/2 activity and WNT5A expression levels were defined as BRAFi‐resistant and named HTB63‐R, A375‐R and A2058‐R, respectively.

### 2.3 Cell viability

The MTT assays were employed to study the cell viability. Approximately 12 000 cells per well of HTB63, HTB63‐R, A375, A375‐R, A2058 and A2058‐R cells were treated with increasing concentrations (0.1, 0.5, 1, 5, 10 μm) of *BRAF* inhibitors, for example, PLX4032 or PLX4720 (Selleckchem, Cat# S1152) for 72 h. In an independent experiment, HTB63‐R cells were incubated with DMSO or the Cdc42‐GTPase inhibitor ML141 (Surviladze *et al*., 2010) at different concentrations (0.25, 0.5, 1, 5, 10 μm) for 48 h. After each treatment, MTT assays were performed as described in the supplementary methods.

### 2.4 Western blotting

Western blotting was performed in accordance with a previously described protocol (Linnskog *et al*., 2014). Briefly, the blotting assays were conducted using primary goat anti‐WNT5A (R&D Systems; 1 : 100), mouse anti‐ERK1/2 (Cell Signalling, Danvers, MA, USA, 1 : 1000), rabbit anti‐phospho‐ERK1/2 (Cell Signalling, 1 : 1000), rabbit anti‐EGFR (Cell Signalling, 1 : 1000), rabbit anti‐PDGFRβ (Cell Signalling, 1 : 1000) and mouse anti‐β‐actin (Sigma‐Aldrich, Stockholm, Sweden; 1 : 30 000) antibodies and secondary HRP‐conjugated rabbit anti‐goat, goat anti‐mouse or goat anti‐rabbit antibodies (Dako, Santa Clara, CA, USA; 1 : 10 000). MDA‐MB‐468 (MDA‐468) breast cancer cell lysates with or without recombinant WNT5A (rW5A) were used as positive and negative controls for WNT5A expression experiments. The densitometric quantifications of relative protein expression were conducted using image lab software (version 6.0, Bio‐Rad, Hercules, CA, USA).

### 2.5 Transwell migration and invasion assay

Approximately 50 000 cells/insert of parental/BRAFi‐R HTB63, A375, and A2058 cells were used to compare their migratory and invasive capacities. In separate experiments, appropriate controls [NaHCO_3_ buffer, pH ~7 for Box5 (WNT5A antagonisitic peptide, Jenei *et al*., 2009) treatment, IgG_1_ isotype antibody (R&D Systems, Minneapolis, MN, USA, Clone #11711) for IL‐6 Ab (R&D Systems, Clone #6708, MAB206) exposure, DMSO for ML141 treatments, 48 h] were used to compare the Box5 (200 μm) and/or IL‐6 Ab (2 μg·mL^−1^) and ML141 (2.5 μm) treatment effects on the cell migration and invasion of HTB63‐R and A375‐R cells. After each experiment, photographs of cells in the inserts were captured using an inverted light microscope (Nikon TMS), and the numbers of migrated/invaded cells were counted using nih imagej ^®^ (NIH Image J, Bethesda, MD, USA) software. The results are presented as the relative migration/invasion compared with parental or vehicle‐treated cells.

### 2.6 IL‐6 ELISA

The amount of secreted IL‐6 from different untreated BRAFi‐sensitive (HTB63, A375 and A2058), untreated BRAFi‐R (HTB63‐R, A375‐R and A2058‐R) and Box5 (200 μm)‐treated BRAFi‐R (HTB63‐R and A375‐R) melanoma cells was quantified in conditioned low‐serum (1% FBS) cell culture medium using the human IL‐6 ELISA Kit (Invitrogen, Carlsbad, CA, USA) according to the protocol supplied by the manufacturer. Briefly, the experiments were performed in 60‐mm cell culture dishes, and cell culture supernatants were collected and centrifuged at 1000 ***g*** for at least 5 min to eliminate cell debris. All the samples were stored at −80 °C prior to analysis.

### 2.7 Cdc42/Rac1‐GTPase activity assay

Cdc42 or Rac1 activities were evaluated using a Rac1/Cdc42 activation assay combo kit from Cell Biolabs (#STA 404) in accordance with the manufacturer's protocol and as described previously (Prasad *et al*., 2013). Briefly, untreated or treated parental and BRAFi‐R HTB63 and A375 cells were lysed in 300 μL of 1X assay/lysis buffer supplied with the kit. Equal concentrations of samples from each of the cell lysates were used to determine the total amounts of Cdc42 and Rac1 by western blotting.

Pull‐down assays were performed separately to determine the Cdc42 and Rac1 activities using at least 300–500 μg of protein from the remaining cell lysates. GTPγS or GDP (supplied with the assay kit) was mixed with the cell lysates and used as either a positive or negative control, respectively. The active and total Cdc42 and Rac1 levels were assessed separately using the anti‐Cdc42 and anti‐Rac1 mouse antibodies supplied with the kit. Chemiluminescent acquisition of the protein band densities was obtained using the ChemiDoc™ imaging system (Bio‐Rad), and densitometric quantifications of the relative Cdc42‐GTP or Rac1‐GTP expression levels were performed using image lab 3.0 software (Bio‐Rad).

### 2.8 Cytoskeleton staining and immunofluorescence imaging

HTB63‐R and A375‐R cells (approx. 10 000 cells/coverslip) were treated with a combination of Box5 (200 μm) and IL‐6 Ab (2 μg·mL^−1^) or ML141 (2.5 μm) for 48 h. Next, the cells were fixed (15 min, 4% paraformaldehyde), washed with 1X PBS (thrice), blocked (20 min, 2% BSA+0.1% Triton X‐100 in 1X PBS) and stained with Phalloidin‐TRITC (1 : 400 dilutions, 1 h). The cell nuclei were stained with DAPI. Finally, the stained cells were mounted with fluorescent mounting solution (Dako, Cat#S3023) on glass slides and observed under a 63X oil objective using a confocal microscope (LSM 700, Carl Zeiss). The TRITC fluorescence (red) intensity of phalloidin‐stained cells in three separate microscopic fields per treatment was measured in slides from six independent experiments and analysed using the lsm 700 (Zeiss‐ LSM 700, Oberkochen, Germany) microscope software; the results are presented as the mean fluorescence intensity.

### 2.9 MTT assay

After the treatment of the parental and BRAFi‐R melanoma cells with the indicated compounds, the medium from each well were replaced with 100 μL of 0.05% MTT reagent and then incubated at 37 °C for 4 h to allow the formation of formazan crystals. The violet formazan crystals were dissolved in a solution containing 0.2% Nonidet™ P40 in isopropanol, and the colour intensity was measured spectrophotometrically at 570 nm. The IC_50_ concentrations for each molecule were defined using the dose–response curve.

### 2.10 WST‐1 cell proliferation assay

For the WST‐1 cell proliferation assay, approximately 25 000 HTB63‐R or A375‐R cells per well (in a 48‐well plate) were exposed to either vehicle (IgG1 antibody, NaHCO_3_ buffer and DMSO) or a fixed dose of Box5 (200 μm) and IL‐6 Ab (2 μg·mL^−1^) and treated with increasing concentrations (0.5, 1.0, 2.5, 5.0) of PLX4032 for 48 h. After the treatment, WST‐1 cell proliferation reagent (#ab155902, Abcam, Cambridge, UK) was added to each well, and the assay was performed in accordance with the manufacturer's instructions.

### 2.11 Statistical analysis

Statistical analyses were performed with Microsoft Office Excel and graphpad prism (GraphPad Prism, San Diego, CA, USA) 5.0 software. Significant differences between two groups were assessed using unpaired or paired Student's *t*‐tests, as indicated in the figure legends. All multiple group analyses were verified for significance using an ANOVA and the Newman–Keuls multiple comparison test. All tests were performed more than three times, and the results are presented as the mean ± SEM. Differences were considered significant when *P *<* *0.05.

## 3 Results

### 3.1 Acquired BRAF inhibitor resistance in *BRAF* ^*V600E*^ mutant melanoma cells results in significantly elevated IL‐6 secretion

**Figure 1:**
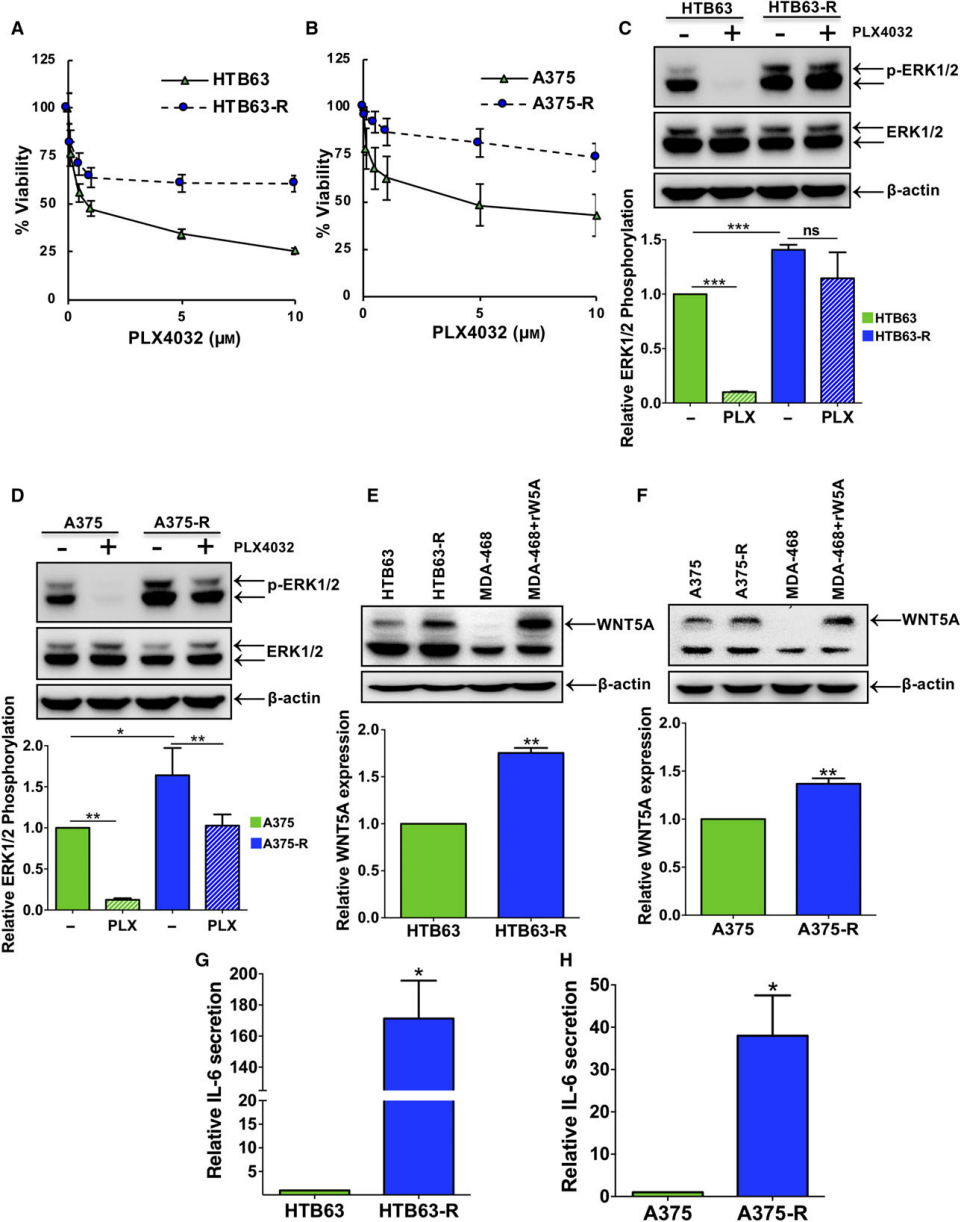
Development of BRAFi resistance in melanoma cells results in a significant increase in IL‐6 secretion. (A) HTB63 (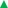)/HTB63‐R (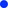) and (B) A375 (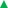)/A375‐R (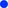) cells were exposed to increasing concentrations of PLX4032. MTT assays were performed as described in the methods section, and graphs were plotted using values from four independent experiments. The results are shown as the mean ± SEM. (C, D) Western blot analyses show the levels of ERK1/2 activity (p‐ERK1/2 protein expression) in PLX4032‐treated parental (HTB63 and A375) and PLX4032‐resistant (HTB63‐R and A375‐R) melanoma cells. β‐Actin was used as a loading control in these experiments. Representative blots from four separate experiments are shown. The graphs represent the densitometric analyses of the ratio between p‐ERK1/2 and ERK1/2 and show p‐ERK1/2 protein expression relative to that of vehicle‐treated nonresistant cells. The results are shown as the mean ± SEM. Statistical analyses were performed using both paired and unpaired Student's *t*‐tests; *P <* *0.05. (E, F) Western blots showing WNT5A protein levels in parental and PLX4032‐resistant HTB63 and A375 cell lines. Representative blots from five separate experiments are shown. The graphs represent the densitometric analyses of WNT5A protein expression normalised against β‐actin, and the results are presented as WNT5A protein expression relative to that of nonresistant cells (*n *=* *5). The results are presented as the mean ± SEM (G, H) IL‐6 secretion from parental and resistant HTB63 and A375 melanoma cells was evaluated by ELISA as described in the methods. The ELISA analyses show secreted IL‐6 protein from parental and PLX4032‐resistant HTB63 (G) and A375 (H) cells. The graphs represent secreted IL‐6 relative to that secreted from nonresistant cells (*n *=* *4). The results are shown as the mean ± SEM. Statistical analyses were performed using unpaired Student's *t*‐tests; *P <* *0.05, **P <* *0.01, ***P <* *0.001.

Here, we established three BRAFi‐R melanoma cell lines through chronic exposure of parental HTB63, A375 and A2058 melanoma cells to the PLX4032 BRAF inhibitor. We observed that PLX4032‐resistant HTB63‐R and A375‐R cells showed a higher IC_50_ (~10 μm) concentration when treated with PLX4032 compared with the parental HTB63 (IC_50_ **= ~1 μm) and A375 (IC_50_ = ~2 μm) cell lines (Fig. 1A,B). We observed that similarly treated A2058‐R cells, despite their intrinsic BRAFi resistance (Boussemart *et al*., 2014), also exhibited a higher IC_50_ value (IC_50_ = ~24.3 μm,* P *<* *0.05) following chronic PLX4032 treatment compared with the parental A2058 cells (IC_50_ = ~20 μm) (Fig. S1A). Based on these observations, we next analysed ERK1/2 activities in parental and BRAFi‐R cells since increased activity of this MAPK has been used as a marker of BRAFi resistance (Su *et al*., 2012). Consistent with these results, we observed increased ERK1/2 activity in HTB63‐R, A375‐R and A2058‐R cells compared with their parental cells (comparing lanes 1 and 3 in Fig. 1C,D and lanes 1 and 2 in Fig. S1B). In accordance with the PLX4032 resistance of BRAFi‐R cells, we found that PLX4032 treatment (24 h) caused an 80% inhibition of ERK1/2 activity in parental HTB63 and A375 cells (comparing lanes 1 and 2 in Fig. 1C,D), whereas it only caused a 30% inhibition of ERK1/2 activity in HTB63‐R and A375‐R cells (comparing lanes 3 and 4 in Fig. 1C,D). We next checked for increased WNT5A expression, which is another established characteristic of BRAFi resistance in melanoma (Anastas *et al*., 2014; O'Connell *et al*., 2013). As expected, we observed an increase in WNT5A expression in all three BRAFi‐R cell lines when compared to that in their parental BRAFi‐sensitive cells (comparing lanes 1 and 2 in Figs 1E,F and S1C). Taken together, the above findings clearly suggested that the established HTB63‐R, A375‐R and A2058‐R cell lines had acquired resistance to BRAF inhibitors. Interestingly, we observed that these HTB63‐R, A375‐R and A2058‐R cells also exhibited resistance to a different BRAF inhibitor (e.g. PLX4720; Fig. S2A–C). We also explored possible changes in the expression of epidermal growth factor receptor (EGFR) and platelet‐derived growth factor receptor beta (PDGFRβ), since these receptors have previously been related to BRAFi resistance in melanomas (Vella *et al*., 2017; Wang *et al*., 2015). Interestingly, we observed that HTB63‐R cells possess significantly increased expression levels of both EGFR and PDGFRβ compared to their parental HTB63 cells (Fig. S3A,B). However, A375‐R melanoma cells only showed a significant increase in the expression of EGFR but not in PDGFRβ levels (Fig. S3C,D).

IL‐6 has been shown to promote invasive migration of parental BRAFi‐sensitive metastatic melanoma cells (Linnskog *et al*., 2014). These observations prompted us to elucidate the effect of acquired BRAFi resistance on the secretion of IL‐6 in the BRAFi‐resistant melanoma cells used in this study. We then made the interesting and novel observation that the amount of IL‐6 secreted from HTB63‐R and A375‐R cells was significantly increased (~170‐ and ~40‐fold, respectively) compared with their parental HTB63 and A375 cells (Fig. 1G,H). We also noticed that IL‐6 secretion from A2058‐R cells was increased more than 2‐fold compared with parental A2058 cells (Fig. S1D). The above results clearly showed that development of acquired BRAFi resistance in melanoma cells was associated with increased IL‐6 secretion from these cells and suggested that it will have significant functional consequences.

### 3.2 Acquired BRAFi‐R of melanoma cells increases their migration and invasion capacities

**Figure 2:**
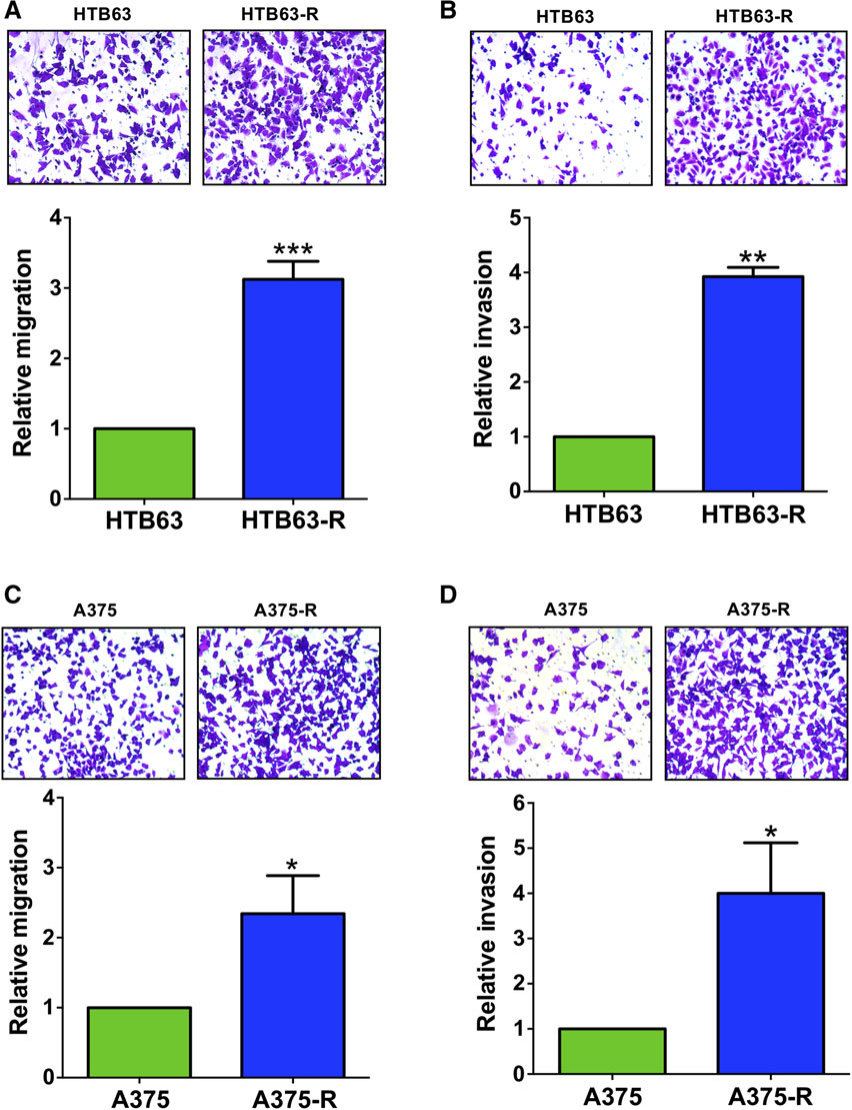
The BRAFi‐R melanoma cells exhibit increased cell migration and invasion. Transwell‐based migration and invasion assays were performed as described in the methods section to compare the cell migration and invasion capacities of parental (HTB63, A375) and PLX4032‐resistant (HTB63‐R, A375‐R) melanoma cells. The migration and invasion of parental and PLX4032‐resistant HTB63 (A, B) and A375 (C, D) cells were quantified by counting the migrated or invaded cells with NIH imagej ^®^ software and are presented as the relative cell migration or invasion. The inserts are photographs of representative experiments showing the number of migrating (A, C) or invading (B, D) parental or resistant cells. The results are calculated in relation to that of nonresistant cells (*n *=* *4) and presented as the mean ± S.E.M. Statistical analyses were performed using unpaired Student's *t*‐tests; *P <* *0.05, **P <* *0.01, ***P <* *0.001.

The increased metastatic behaviour of BRAFi‐R melanoma prompted us to study the effect of acquired BRAFi resistance on the migration and invasion of HTB63‐R, A375‐R and A2058‐R cells. Consistent with the findings of elevated IL‐6 secretion and WNT5A expression, we observed increased migration and invasion of these BRAFi‐R melanoma cells compared with their parental HTB63, A375 and A2058 cells (Figs 2 and S4). These results suggest that elevated secretion of IL‐6 along with increased expression of WNT5A in HTB63‐R, A375‐R and A2058‐R cells could be responsible for mediating the observed increase in migration and invasion of these cells. In the future, it would also be interesting to test whether the increased expression of the tyrosine kinase receptors (EGFR and PDGFRβ) is independent of or in any way related to the enhanced IL‐6 and/or WNT5A signalling and increased migration and invasion during the development of BRAFi resistance in melanoma.

### 3.3 The increase in IL‐6 secretion and WNT5A expression are independent events in BRAFi‐R melanoma cells

**Figure 3:**
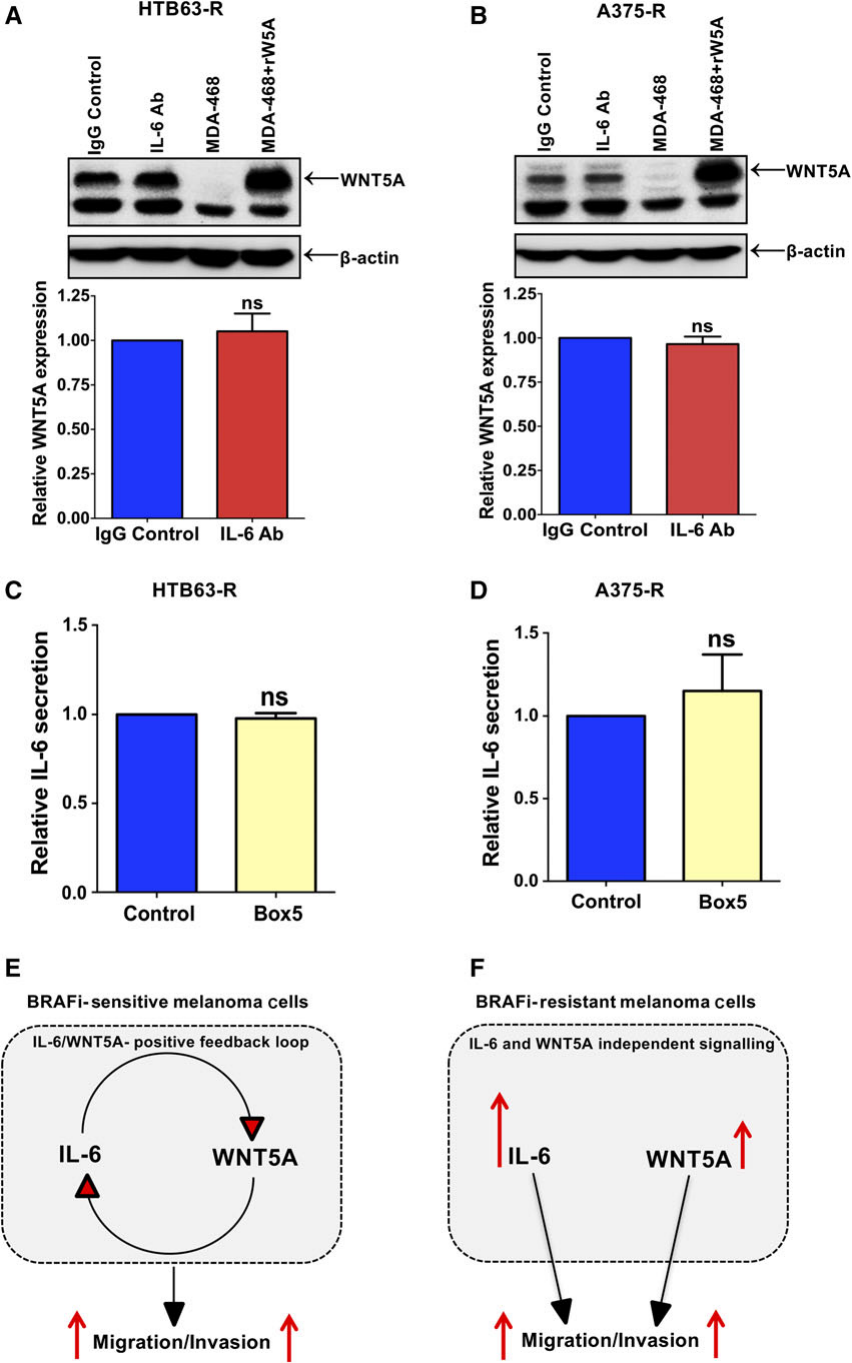
Increased IL‐6 secretion and WNT5A expression are independent events in BRAFi‐R melanoma cells. Western blot analyses showing WNT5A protein expression in IL‐6 Ab‐treated HTB63‐R (A) and A375‐R (B) melanoma cells. The graphs represent densitometric analyses of WNT5A expression normalised against β‐actin from six separate experiments. The results are presented as WNT5A expression in relation to that of nonresistant cells (*n *=* *6) and given as the mean ± SEM. Statistical analyses were performed using paired Student's *t*‐tests. (C, D) IL‐6 secretion from Box5‐treated HTB63‐R and A375‐R melanoma cells was evaluated by ELISA as described in Materials and Methods. The ELISA analyses show the secreted IL‐6 levels from Box5‐treated HTB63‐R (C) and A375‐R (D) cells (*n *=* *6). The results on IL‐6 secretion are presented in relation to that of nonresistant cells and given as the mean ± SEM. Statistical analyses were performed using paired Student's *t*‐tests. (E, F) Cartoons outlining the IL‐6/WNT5A positive feedback loop in (E) BRAFi‐sensitive melanoma cells and the independent IL‐6 and WNT5A signalling in (F) BRAFi‐R melanoma cells and how they affect invasive migration in these cells.

We have previously reported the existence of an IL‐6/WNT5A positive feedback loop in parental BRAFi‐sensitive melanoma cells that is related to their migratory and invasive properties (Linnskog *et al*., 2016). The above findings of a parallel increase in WNT5A expression and IL‐6 secretion are in good agreement with such an IL‐6/WNT5A feedback loop also in BRAFi‐R melanoma cells. To investigate this hypothesis, we first treated HTB63‐R and A375‐R cells with an IL‐6 Ab and analysed the expression of WNT5A (Fig. 3A,B). In a separate set of experiments, we exposed these BRAFi‐R cells to the WNT5A antagonistic peptide Box5 and quantified the secretion of IL‐6 (Fig. 3C,D). However, because the BRAFi‐R melanoma cells secreted much more IL‐6 and expressed more WNT5A, we increased the dose of IL‐6 Ab and Box5 peptide to 2 μg·mL^−1^ and 200 μm, respectively. Surprisingly, we observed no effect of IL‐6 Ab treatment on WNT5A expression (Fig. 3A,B), nor did we find an inhibition of Box5 treatment on IL‐6 secretion (Fig. 3C,D) from these BRAFi‐R melanoma cells. Our results suggested that the molecular mechanism underlying the IL‐6/WNT5A feedback loop in parental melanoma cells was lost during the development of acquired BRAFi resistance in melanoma cells, as schematically outlined in Fig. 3E,F. The question then arises concerning if and how IL‐6 and WNT5A will affect migration and invasion of BRAFi‐R melanoma cells in the absence of an IL‐6/WNT5A positive feedback loop.

### 3.4 Inhibition of IL‐6 or WNT5A signalling as well as combined impaired signalling inhibit cell migration and invasion of BRAFi‐R melanoma cells

**Figure 4:**
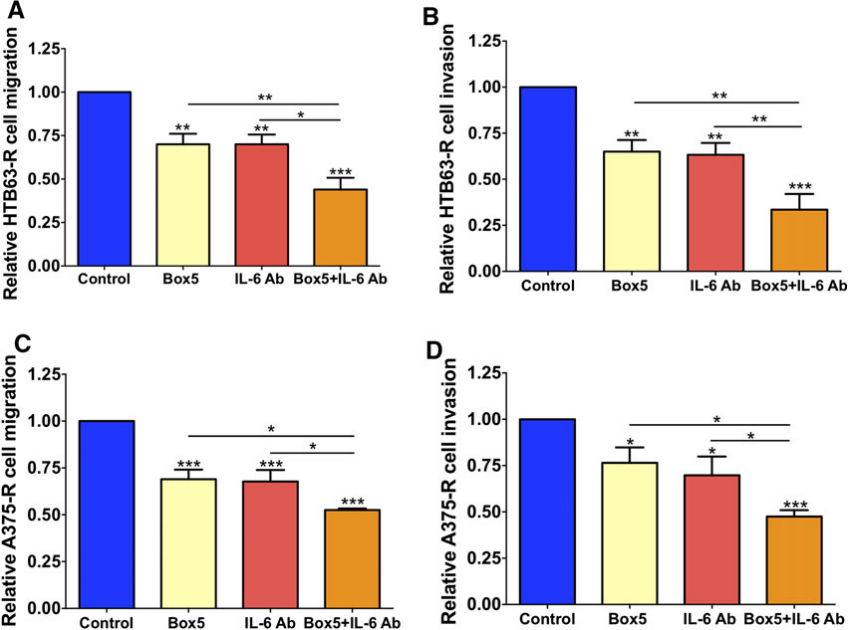
Combined treatment with an anti‐IL‐6 Ab and the WNT5A antagonist Box5 more effectively impairs the migration and invasion of BRAFi‐R melanoma cells. (A, B) HTB63‐R and (C, D) A375‐R cells were exposed to an IL‐6 Ab and/or Box5. Their migratory and invasive responses were evaluated as described in the methods. Cells were pretreated with vehicle, IL‐6 Ab or/and Box5 for 48 h, after which migration/invasion assays were initiated and performed for 24 h in the presence of freshly added vehicle, IL‐6 Ab or/and Box5. After the assays were completed, the numbers of migrating or invading cells were quantified by counting the cells with NIH imagej ^®^ software and are shown as the relative cell migration or invasion. The results (*n *=* *4) are given in relation to that of vehicle‐treated control cells (*n *=* *4) and given as the mean ± SEM. Statistical analyses were performed using ANOVA and the Newman–Keuls multiple comparison test; *P <* *0.05, **P <* *0.01, ***P <* *0.001.

To evaluate the independent and combined effects of IL‐6 and WNT5A signalling on the invasive migration of BRAFi‐R melanoma cells, we first blocked WNT5A or IL‐6 signalling separately in HTB63‐R and A375‐R cells by using either Box5 or an IL‐6 Ab. We noticed that individual exposure to Box5 or an IL‐6 Ab caused reduced migration and invasion of HTB63‐R and A375‐R melanoma cells by 25–30% in comparison with their vehicle controls (Fig. 4A–D). Next, we combined Box5 and IL‐6 Ab treatment to evaluate the therapeutic significance of inhibiting these independent factors simultaneously. Interestingly, we observed that the combination of Box5 and the IL‐6 Ab reduced the migration and invasion of both HTB63‐R and A375‐R cells by approximately 50% or more in comparison with their vehicle‐treated control cells (Fig. 4A–D). The results clearly showed that direct and simultaneous inhibition of these two independent signalling events was a more effective approach to inhibit the invasive migration of BRAFi‐R melanoma cells. Due to the practical limitations of migration and invasion assays, we could not determine conclusively whether the combined antimigratory/invasive effect of IL‐6 Ab and Box5 treatment on BRAFi‐R melanoma cells was additive or synergistic. However, from the result presented in Fig. 4 showing the individual and combined effects of Box5 and/or IL‐6 Ab on the migration and invasion of HTB63‐R and A375‐R cells, one might speculate that the combined inhibition of these independent components (IL‐6 and WNT5A) has an additive effect on reducing the invasive migration of these BRAFi‐R melanoma cells. Since IL‐6 and WNT5A signalling independently promoted invasive migration and their combined inhibition more effectively reduced the migration and invasion of HTB63‐R and A375‐R melanoma cells, we speculated that the simultaneous inhibition of these two signalling pathways decreased the invasive migration via altered downstream signalling and modulation of the actin cytoskeleton.

### 3.5 Combined exposure to a blocking IL‐6 Ab and the WNT5A antagonist Box5 inhibits the elevated Cdc42‐GTPase activity of BRAFi‐R melanoma cells

**Figure 5:**
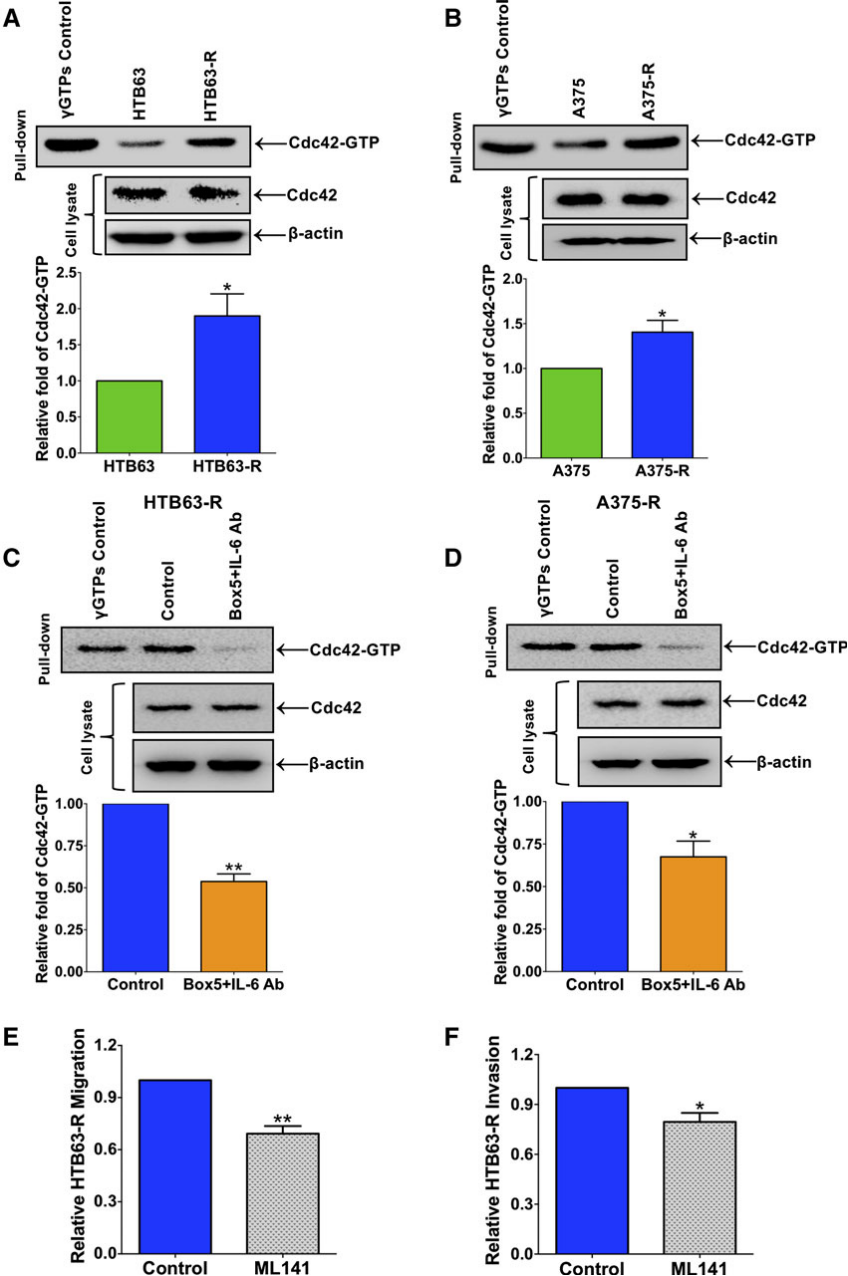
Simultaneous inhibition of IL‐6 and WNT5A signalling reduces the invasive migration of BRAFi‐R melanoma cells through the inhibition of Cdc42‐GTPase activity. First, active Cdc42 levels were determined in parental and resistant HTB63 (A) and A375 (B) cells through a pull‐down assay using GST‐PAK1‐PBD beads as described in the methods. The blots outline representative experiments showing the levels of Cdc42‐GTP in the pull‐down and the total amounts of Cdc42 and β‐actin in the cell lysates. The graphs represent the densitometric analyses of the Cdc42‐GTP protein bands in resistant cells relative to parental cells. Next, HTB63‐R (C) and A375‐R (D) cells were treated with the combination of an IL‐6 Ab and Box5, and Cdc42‐GTP levels were analysed through pull‐down assays as mentioned in Materials and Methods. The blots outline representative experiments showing the levels of Cdc42‐GTP that had been pulled down and the total amounts of Cdc42 and β‐actin in the cell lysates. The graphs represent densitometric analyses of the Cdc42‐GTP protein band in IL‐6 Ab and Box5‐treated cells relative to cells treated with vehicle. (E, F) HTB63‐R cells were pretreated with vehicle or ML141 for 48 h, the cells were washed and transferred to the inserts of the transwell migration or invasion plates, and the assays were performed for 24 h as described in Materials and Methods. The effects of the ML141 treatment on migration and invasion are shown relative to that of vehicle‐treated (DMSO) HTB63‐R cells. All the results in this figure are derived from separate experiments and are shown as the mean ± S.E.M. Statistical analyses were performed using (A, B) unpaired and (C–F) paired Student's *t*‐tests; *P <* *0.05, **P <* *0.01.

The small GTPase Cdc42 has been reported to be an important regulator of melanoma cell invasion (Gadea *et al*., 2008). However, in BRAFi‐R melanoma cells, no data are available regarding possible altered Cdc42/Rac1‐GTPase activities. Based on this deficiency and our present findings that BRAFi‐R melanoma cells exhibit increased migration and invasion (Fig. 2), we investigated Cdc42 activities in these BRAFi‐R cells. Interestingly, we found that Cdc42‐GTPase activity (referred to as Cdc42‐GTP in the figures) was dramatically elevated in both HTB63‐R (Fig. 5A) and A375‐R (Fig. 5B) cells. We also observed an increase in Rac1 activity (referred to as Rac1‐GTP) in HTB63‐R cells (Fig. S5A). Next, we investigated whether combined inhibition of both IL‐6 and WNT5A signalling had any effect on Cdc42‐GTPase or Rac1‐GTPase activity in BRAFi‐R melanoma cells. Interestingly, we documented significantly reduced Cdc42‐GTP levels in HTB63‐R (Fig. 5C) and A375‐R (Fig. 5D) cells treated with the combination of IL‐6 Ab and Box5 compared with the vehicle‐treated controls. However, we did not observe a significant reduction in Rac1‐GTPase activity in HTB63‐R cells treated with the combination of IL‐6 Ab and Box5 (Fig. S5B), arguing against an essential role of Rac1 in the IL‐6‐ and WNT5A‐dependent invasive migration of BRAFi‐R melanoma cells. To confirm that Cdc42‐GTPase could promote migration and invasion in BRAFi‐R melanoma cells, we exposed HTB63‐R cells to a specific‐Cdc42‐GTPase inhibitor, for example ML141 (Surviladze *et al*., 2010). First, we defined a nontoxic concentration of ML141 using the MTT assay since a toxic effect would nonspecifically reduce cell migration and invasion. We found that 2.5 μm of ML141 had no toxic effect on HTB63‐R cells (Fig. S6). Consistent with an important role of Cdc42‐GTPase in regulating the invasive migration of BRAFi‐R melanoma cells, ML141 treatment caused a significant reduction of the migration and invasion of the HTB63‐R cells compared with the vehicle‐treated control cells (Fig. 5E,F). The above observations suggested that Cdc42‐GTPase activity was essential for the increased migration and invasion of BRAFi‐R melanoma cells and that the combined inhibition of IL‐6 and WNT5A signalling in these cells reduced their invasive migration via diminished Cdc42‐GTPase activity.

### 3.6 Combined exposure to an IL‐6 Ab and Box5 modulates the organisation and reduces the content of filamentous actin in BRAFi‐R melanoma cells

**Figure 6:**
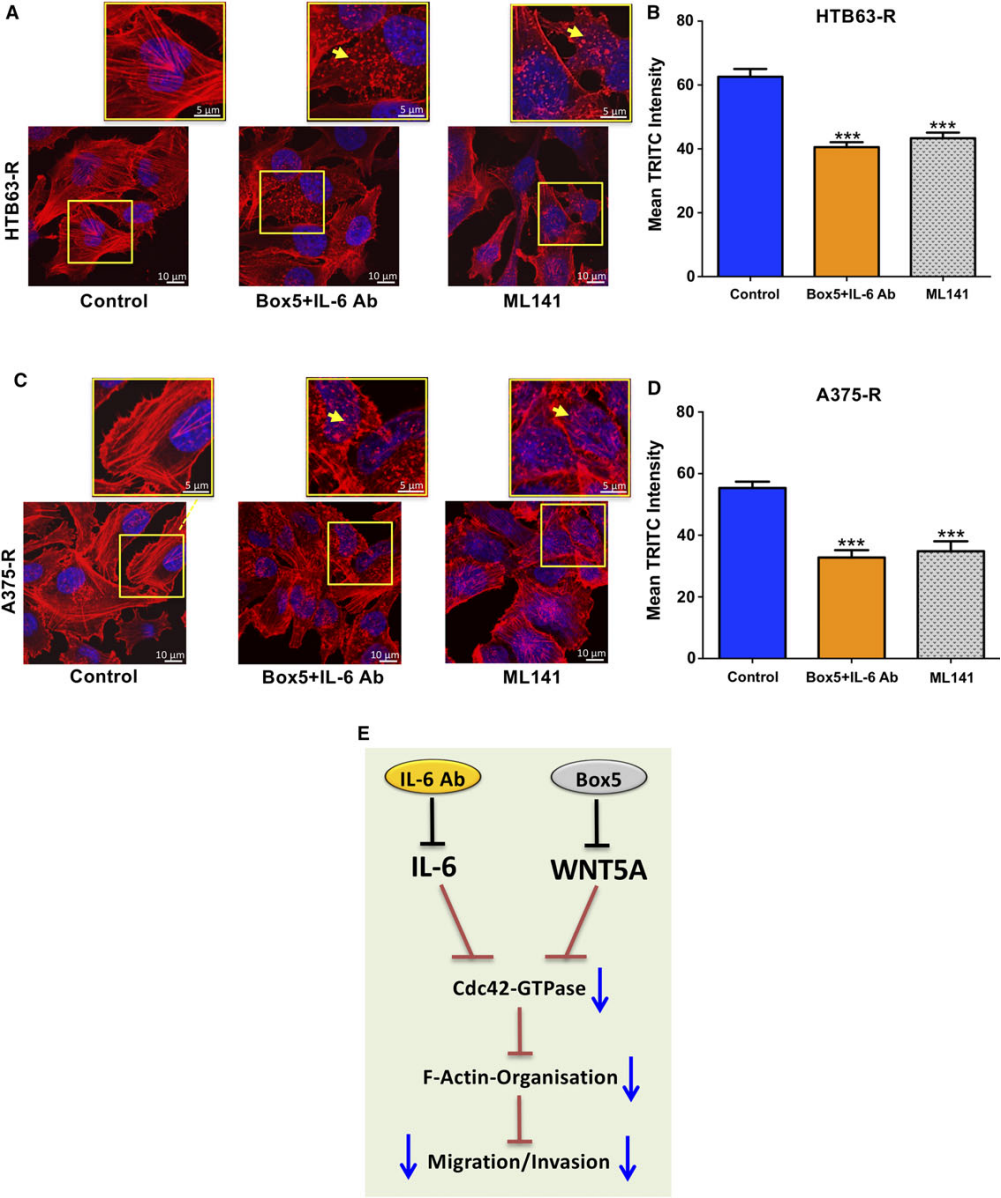
Effects of a combination of an IL‐6 Ab and Box5 or ML141 on the F‐actin network in BRAFi‐R melanoma cells. (A, B) HTB63‐R and (C, D) A375‐R cells were treated with Box5 plus an IL‐6 Ab or ML141 for 48 h, and F‐actin staining was performed as described in the Methods section. The representative photomicrographs show the effects of these treatments on the F‐actin network in HTB63‐R and A375‐R cells. The scale bars represent 10 μm in the larger images. The regions of interest (marked with yellow boxes) are further magnified and placed above each original image. The scale bars in these magnified images represent 5 μm. The yellow arrows in these smaller and magnified images indicate disrupted/disarranged actin filaments in BRAFI‐R melanoma cells. The graphs represent the mean TRITC (red) fluorescence intensity quantified from six independent experiments as mentioned in the methods section. The results are shown as the mean ± SEM. Statistical analyses were performed using ANOVA and the Newman–Keuls multiple comparison test; ***P <* *0.001. (E) Cartoon summarising the effects of combined treatment with a neutralising IL‐6 Ab and the WNT5A antagonist Box5 on migration/invasion of BRAFi‐R melanoma cells.

The Cdc42‐GTPase is among the most studied GTPases and is considered essential in the organisation of the cellular actin network and tumour cell motility (Stengel and Zheng, 2011; Xiao *et al*., 2018). Based on our previous results that the combination of an IL‐6 Ab and Box5 reduced both Cdc42‐GTPase activity and the invasive migration of BRAFi‐R melanoma cells, we next investigated whether inhibition of Cdc42‐GTPase activity could affect the level of filamentous actin (F‐actin) in these resistant cells and thus explain their impaired invasive migration. Confocal microscopy analyses of F‐actin staining with phalloidin‐TRITC revealed that direct and simultaneous inhibition of IL‐6 and WNT5A signalling resulted in disorganised and less dense F‐actin structures in both HTB63‐R (Fig. 6A,B) and A375‐R (Fig. 6C,D) cells compared with those in untreated control cells. To confirm that Cdc42‐GTPase is involved in the alterations of the F‐actin network observed above, we treated BRAFi‐R cells with the Cdc42‐GTPase inhibitor ML141. The ML141 inhibitor clearly mimicked the effect of the simultaneous inhibition of IL‐6 and WNT5A signalling on the F‐actin organisation and content (Fig. 6A–D). Taken together, the present findings demonstrated that the combined inhibition of IL‐6 and WNT5A signalling in BRAFi‐R melanoma cells effectively decreased their invasive migration via the reduction of Cdc42‐GTPase activity and subsequent re‐organisation of the actin network and decreased F‐actin content (as schematically outline in Fig. 6E). This study focused on preventing the increased invasive migration of BRAFi‐R melanoma cells; however, our combined treatment with an IL‐6 antibody and Box5 also restored the sensitivity of these cells to the BRAF inhibitor PLX4032 (Fig. S7A–D). We used a highly sensitive WST‐1 cell proliferation assay to assess the antiproliferative effect of BRAFi treatment following direct inhibition of IL‐6 and WNT5A signalling in HTB63‐R and A375‐R BRAFi‐R melanoma cell lines. Interestingly, the IC_50_ dose of PLX4032 was decreased by at least 60% in HTB63‐R cells (Fig. S7A,B) and 35% in A375‐R cells (Fig. S7C,D) following treatment with the combination of an IL‐6 Ab and Box5.

## 4 Discussion

The BRAFi‐R melanomas are linked to increased metastatic behaviour (O'Connell *et al*., 2013), presumably via the increased migration and invasion of tumour cells. Therefore, in the present study, we investigated how BRAFi‐R melanoma cell migration and invasion are regulated. Importantly, we observed a previously unrecognised substantial increase in IL‐6 secretion from BRAFi‐R melanoma cells paralleling the elevated invasive migration of these cells. Our observation of elevated IL‐6 secretion is in accordance with the observation that IL‐6 is not only a promoter of melanoma metastasis (Na *et al*., 2013) but also an inducer of WNT5A protein expression in parental BRAFi‐sensitive melanoma cells (Linnskog *et al*., 2014). Furthermore, an IL‐6/WNT5A positive feedback loop has been previously shown to be associated with the increased invasive migration of parental BRAFi‐sensitive melanoma cells (Linnskog *et al*., 2016). However, in contrast to this latter finding, we found that the IL‐6/WNT5A positive feedback loop is absent in BRAFi‐R melanoma cells. This conclusion is based on our findings that IL‐6 blockade did not decrease the expression of WNT5A nor did inhibition of WNT5A signalling reduce the IL‐6 secretion from BRAFi‐R cells. These results clearly indicate that any functional effect of IL‐6 on BRAFi‐R melanoma cells is independent of WNT5A and that the cellular context is crucial for the ability of IL‐6 or WNT5A to stimulate WNT5A expression and IL‐6 secretion, respectively.

Only a small number of studies have examined a possible association between IL‐6 expression and IL‐6 secretion with acquired or inherent drug resistance in cancer. In one such study, it was reported that basal IL‐6 levels were higher in astrocytoma xenografts with resistance to selumetinib (a MEK1/MEK2 inhibitor) than in parental selumetinib‐sensitive astrocytoma xenografts (Bid *et al*., 2013). However, since resistance to selumetinib in these tumour xenografts was also reported to be unstable, its relation to IL‐6 levels is uncertain. In another study, IL‐6 levels were higher in thyroid cancer cell lines with inherent apoptosis resistance to RAF‐MAPK inhibitors than RAF‐MAPK inhibitor‐sensitive melanoma and colon cancer cell lines (Sos *et al*., 2014). However, the authors of these studies did not explore the relationship of IL‐6 levels with tumour cell migration and invasion. Based on our present finding that IL‐6 and WNT5A independently mediate their effects on the invasive migration of BRAFi‐R melanoma cells, we investigated whether and how elevated IL‐6 secretion and WNT5A expression translated into increased BRAFi‐R melanoma cell migration and invasion.

To investigate the importance of IL‐6 and WNT5A signalling for the increased invasive migration of BRAFi‐R melanoma cells, we separately or in combination blocked these signalling molecules. When BRAFi‐R melanoma cells were exposed separately to either an IL‐6 Ab or the WNT5A antagonist Box5, it caused, in both cases, impaired invasive migration of these cells. However, when these two approaches were combined, the effect on invasive migration was more pronounced, which is consistent with the above conclusion that the ability of IL‐6 to promote invasive migration of BRAFi‐R melanoma cells is independent of WNT5A.

Cancer cell migration and invasion are dependent on dynamic changes in their actin cytoskeleton (Yamaguchi and Condeelis, 2007). In search of regulators of the cytoskeleton in BRAFi‐R melanoma cells, we found that the induction of BRAFi resistance increases the activity of the small GTPase Cdc42 in melanoma cells. These findings are in good agreement with a previous study demonstrating that the expression of Cdc42 in melanoma tumour tissue was positively correlated with melanoma metastasis (Tucci *et al*., 2007). To investigate the importance of this signalling molecule, we next blocked IL‐6 and WNT5A signalling as well as Cdc42‐GTPase activation itself in BRAFi‐R melanoma cells and studied how these treatments affected the cellular actin network. Our data indicate that combined treatment with an IL‐6 Ab and Box5 efficiently reduced Cdc42‐GTPase activity and decreased F‐actin content in these cells. Although we observed an increase in Rac1 activity in BRAFi‐R melanoma cells, we did not observe a statistically significant reduction in the elevated Rac1 activity of BRAFi‐R melanoma cells after combined treatment with an IL‐6 Ab and Box5. Thus, we can exclude an essential role of Rac1 in the regulation of invasive migration of BRAFi‐R melanoma cells. These data are in partial agreement with the findings presented in a study in which Box5 treatment reversed WNT5A‐mediated activation of both Cdc42 and Rac1 in parallel with a reduction of human corneal endothelial cell migration (Lee and Heur, 2014). However, our data are in contrast with a report on gastric cancer cells in which the authors suggest that reduced Rac1 activity explains why a WNT5A antibody impairs the migration and invasion of these cells (Hanaki *et al*., 2012). Based on our present observations, we conclude that simultaneous inhibition of IL‐6 and WNT5A signalling effectively obstruct Cdc42‐dependent migration and invasion of BRAFi‐R melanoma cells.

Previously, a proof of principle for the idea that WNT5A mediates BRAFi‐R was obtained by siRNA knockdown of either WNT5A or its putative receptors Frizzled‐7 and ROR2 (Anastas *et al*., 2014; O'Connell *et al*., 2013). Although not the primary focus of this study, we believe that using a blocking IL‐6 Ab and the WNT5A antagonist Box5, which will also restore the sensitivity of BRAFi‐R melanoma cells to BRAFi treatment, is a more relevant therapeutic approach from our perspective.

Altogether, our study demonstrates that the development of BRAFi‐R in melanoma cells triggers a significant increase in IL‐6 secretion along with elevated WNT5A protein expression. However, the IL‐6/WNT5A positive feedback loop present in parental melanoma cells is lost during development of acquired BRAFi resistance in melanoma cells. Our present study suggests a possible therapeutic potential for a future IL‐6/WNT5A‐based antimetastatic approach for BRAFi‐R melanoma patients.

## 5 Conclusions

In the present study, we have shown for the first time that the development of acquired resistance to BRAFi causes a significant increase in IL‐6 secretion in BRAF‐mutant melanoma cells. Furthermore, in contrast to the parental BRAFi‐sensitive melanoma cells, IL‐6 and WNT5A signalling independently promote the invasive migration of BRAFi‐R melanoma cells. Finally, our finding that the simultaneous inhibition of IL‐6 and WNT5A signalling by the combination of an IL‐6 antibody and the WNT5A antagonistic peptide Box5 efficiently impairs the invasive migration of BRAFi‐R melanoma cells by impairing their elevated Cdc42‐GTPase activity, forms the basis for a possible novel antimetastatic therapeutic option for BRAFi‐R melanoma patients.

## Author contributions

PM and TA conceived the idea of the study. PM performed all the experiments in the study and wrote the first draft of the manuscript. CP provided critical suggestions for the Cdc42‐activation experiments. PM and TA discussed the results and interpreted the data. TA, PM and CP critically reviewed and corrected the manuscript. All authors reviewed and agreed to the information provided in this manuscript.

## Conflict of interest

TA is a shareholder of WntResearch and is the part‐time Chief Scientific Officer of WntResearch. This does not alter the author's adherence to all guidelines for publication in Molecular Oncology.

## Supporting information


**Fig. S1**. Acquired BRAFi resistance in A2058 melanoma cells results in a significant increase in IL‐6 secretion.
**Fig. S2**. PLX4032‐resistant melanoma cells are also resistant to PLX4072 BRAF inhibitor.
**Fig. S3**. Acquired BRAFi resistance in melanoma cells affects EGFR and PDGFRβ expression.
**Fig. S4**. BRAFi‐R A2058 melanoma cells exhibit increased cell migration and invasion.
**Fig. S5**. Effect of combined inhibition of IL‐6 and WNT5A signalling on Rac1‐GTPase activity.
**Fig. S6**. Toxicity dose determination of a Cdc42 inhibitor in HTB63‐R cells.
**Fig. S7**. Direct inhibition of IL‐6 and WNT5A signalling efficiently restores the sensitivity of BRAFi‐R cells to PLX4032.
