# Supplementary material for: Combination therapy targeting the elevated interleukin‐6 level reduces invasive migration of BRAF inhibitor‐resistant melanoma cells
Source: Mol Oncol. 2019 Jan 10;13(2):480–94. doi: 10.1002/1878-0261.12433 (PMC6360505; doi:10.1002/1878-0261.12433)
Supplement: Supplementary file 1 — Fig. S1. Acquired BRAFi resistance in A2058 melanoma cells results in a significant increase in IL‐6 secretion. Fig. S2. PLX4032‐resistant melanoma cells are also resistant to PLX4072 BRAF inhibitor. Fig. S3. Acquired BRAFi resistance in melanoma cells affects EGFR and PDGFRβ expression. Fig. S4. BRAFi‐R A2058 melanoma cells exhibit increased cell migration and invasion. Fig. S5. Effect of combined inhibition of IL‐6 and WNT5A signalling on Rac1‐GTPase activity. Fig. S6. Toxicity dose determination of a Cdc42 inhibitor in HTB63‐R cells. Fig. S7. Direct inhibition of IL‐6 and WNT5A signalling efficiently restores the sensitivity of BRAFi‐R cells to PLX4032. [file MOL2-13-480-s001.docx]

**Supplementary Information**

**Combination therapy targeting the elevated interleukin-6 level reduces invasive migration of BRAF inhibitor-resistant melanoma cells**

Purusottam Mohapatra, Chandra Prakash Prasad, Tommy Andersson

**Supplemental Figure 1. Acquired BRAFi resistance in A2058 melanoma cells results in a significant increase in IL-6 secretion.**

A

B


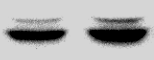

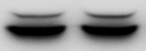

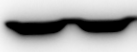


**A2058**

**A2058-R**

**β-actin**

**ERK1/2**

**p-ERK1/2**

←

←

←

←

←

C

D

**MDA-468+rW5A**

**MDA-468**

**A2058**

**A2058-R**


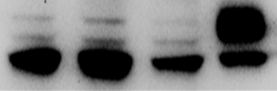

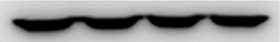


**β-actin**

**WNT5A**

←

←


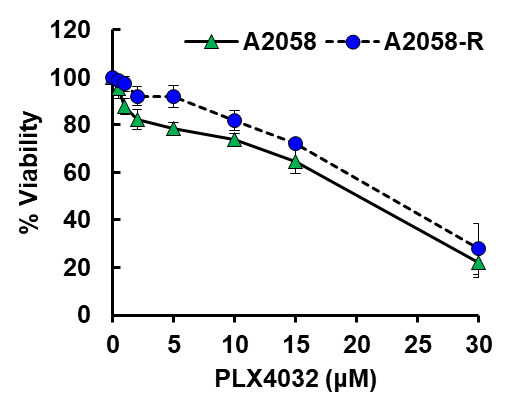


**Figure S1.** Cell viability of (**A)** A2058 (▲)/ A2058-R (●) cells treated with PLX4032 was evaluated with MTT assays as described in the main Methods section. The results (n=4) are presented as the mean ± S.E.M. (**B)** Western blot analysis showing the protein levels of endogenous ERK1/2 and p-ERK1/2 in A2058 and A2058-R cells. The graph represents the densitometric analyses of the ratio of p-ERK1/2 and ERK1/2 and shows the relative p-ERK1/2 protein expression. The results (n=4) are presented as the mean ± S.E.M. Statistical analyses were performed using unpaired t-tests; *, p < 0.05. **(C)** Western blot analyses of the protein levels of endogenous WNT5A in parental and PLX4032-resistant A2058 cells. β-actin was used as a loading control. The graph represents the densitometric analyses of WNT5A protein expression normalised against β-actin, and the results are presented as the relative WNT5A protein expression. (**D)** IL-6 secretion from A2058 and A2058-R cells were evaluated by ELISAs as described in the main Methods section. The results shown in the graphs (n=4) are presented as the mean ± S.E.M. Statistical analyses were performed using unpaired t-tests; *, p < 0.05.

**Supplemental Figure 2. PLX4032-resistant melanoma cells are also resistant to PLX4072 BRAF inhibitor.**

A


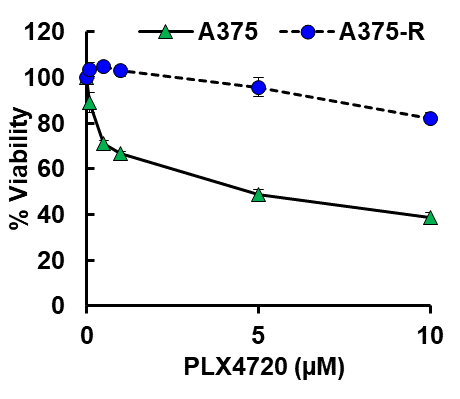


B


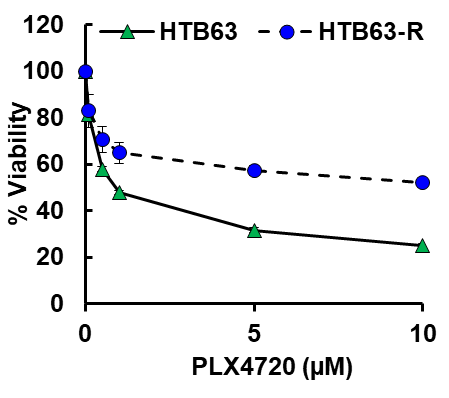

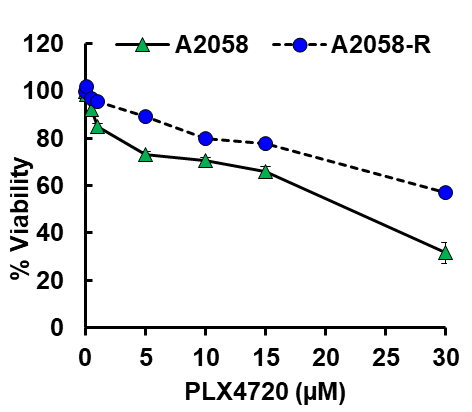


C

**Figure S2.** Cell viability of (**A)** HTB63 (▲)/HTB63-R (●), (**B)** A375 (▲)/A375-R (●), (**C) and** A2058 (▲)/A2058-R (●) cells treated with different doses of PLX4720 were evaluated with MTT assays (n=3) as described in the main Methods section. The results in all panels are presented as the mean ± S.E.M.

**Supplemental Figure 3. Acquired BRAFi-resistance in melanoma cells affects EGFR and PDGFRβ expression.**

B


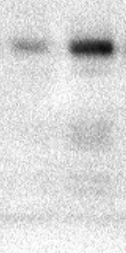


**PDGFRβ**

←


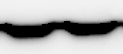


**β-actin**

←

**HTB63**

**HTB63-R**

A


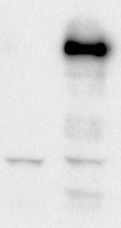


**EGFR**

←


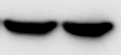


**β-actin**

←

**HTB63**

**HTB63-R**

C


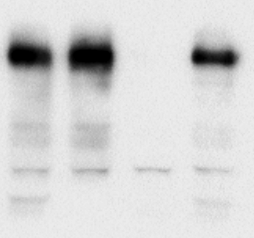


**EGFR**

←


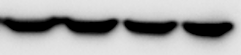


**β-actin**

←

**A375**

**A375-R**

D


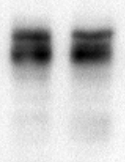


**PDGFRβ**

←


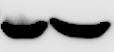


**β-actin**

←

**A375**

**A375-R**

**Figure S3.** Western blot analysis show the expressions of (A, C) EGFR and (B, D) PDGFRβ in parental and BRAFi-R HTB63 and A375 cells. β-actin was used as a loading control. The graphs represent the densitometric analyses of EGFR or PDGFRβ protein expression normalised against β-actin, and the results are presented as relative EGFR or PDGFRβ protein expression. The results (n=4) are presented as means ± S.E.M. Statistical analyses were performed using unpaired Student’s t-tests; *, p < 0.05; **, p < 0.01.

**Supplemental Figure 4. BRAFi-R A2058 melanoma cells exhibit increased cell migration and invasion.**

A

B

**Figure S4.** Transwell-based migration and invasion assays were performed as described in the Methods section to compare the cell migration/invasion efficiency of A2058 and A2058-R cells. The cell **(A)** migration and (**B)** invasion of A2058/A2058-R cells were quantified by counting the migrated/invaded cells using NIH ImageJ software and are presented as the relative cell migration/invasion. The results in both panels (n=4) are presented as the mean ± S.E.M. Statistical analyses were performed using unpaired t-tests; *, p < 0.05; **, p < 0.01.

**Supplemental Figure 5. Effect of combined inhibition of IL-6 and WNT5A signalling on Rac1-GTPase activity.**

A

B

**HTB63**

**HTB63-R**

**γGTPs Control**

Pull-down

**Rac1-GTP**

**Rac1**

**β-actin**


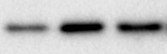

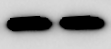

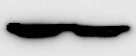


Pull-down

**Control**

**γGTPs Control**

**Box5+IL-6 Ab**

**Rac1-GTP**

**Rac1**

**β-actin**


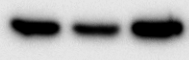

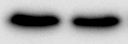

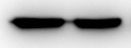


Cell lysate

Cell lysate

←

←

←

←

←

←

HTB63-R

**Figure S5.** The active-Rac1 (Rac1-GTP) levels were determined in **(A)** HTB63 and HTB63-R cells or **(B)** Box5 and IL-6 Ab-treated HTB63-R cells through pull-down assays using GST-PAK1-PBD beads as described in the main Methods section. The graphs represent the quantified protein band density of Rac1-GTP and are presented as the relative Rac1-GTP levels compared with those in parental cells. The results in the graphs (n=4) are presented as the mean ± S.E.M. Statistical analyses were performed using (A) unpaired and (B) paired t-tests; *, p < 0.05; **, p < 0.01.

**Supplemental Figure 6. Toxicity dose determination of a Cdc42 inhibitor in HTB63-R cells.**

**Figure S6.** The cell viability of HTB63-R cells treated with different doses of ML141 (Cdc42-GTPase-specific inhibitor) was evaluated with MTT assays (n=3) as described in the main Methods section. The results are presented as the mean ± S.E.M.

**Supplementary Figure 7. Direct inhibition of IL-6 and WNT5A signalling efficiently restores the sensitivity of BRAFi-R cells to PLX4032.**

A

B

HTB63-R

A375-R

C

D

HTB63-R

A375-R


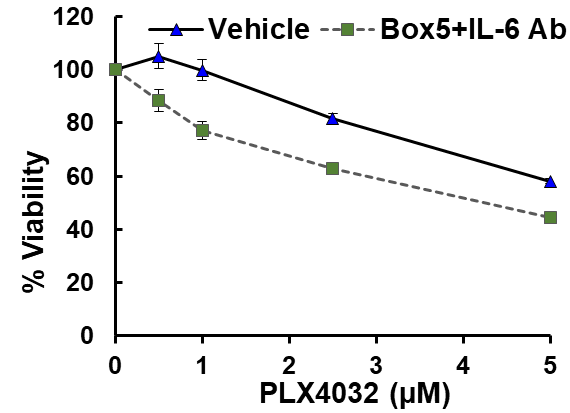

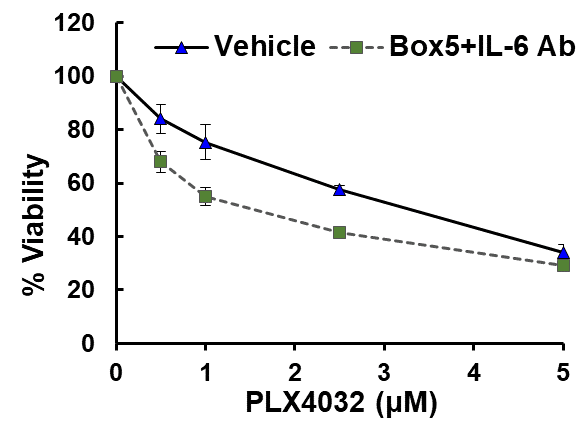


**Figure S7.** WST-1 cell proliferation assays were performed to evaluate the sensitivity of Box5 and IL-6 Ab-treated HTB63-R and A375-R cells to PLX4032. (**A, B**) HTB63-R and (**C, D**) A375-R cells treated with vehicle (▲) or the combination of IL-6 Ab and Box5 (■) were exposed to increasing concentrations of PLX4032 for 48 hrs, and WST-1 cell proliferation assays were performed as described in the Methods section. The histogram shows the comparative analyses of relative *IC_50_* values of PLX4032 between vehicle-treated *vs* IL-6 Ab and Box5-treated HTB63-R (**B**) and A375-R (**D**) cells. The results (n=4) are shown as the mean ± S.E.M. Statistical analyses were performed using paired Student’s *t*-tests; *, p < 0.05, **, p < 0.01.
